# Dielectric Properties and Electromagnetic–Thermal–Moisture Coupling of Frozen Soil Under Microwave Irradiation

**DOI:** 10.3390/ma19122583

**Published:** 2026-06-15

**Authors:** Baoyi He, Zixin He, Zhuo Chen, Yixiang Zhang, Hongge Han, Yu Li, Zihan Li, Litao Zhao, Anshuai Wang, Xuehui Yu

**Affiliations:** 1 Xi’an Key Laboratory of Mechanics of Building Materials, School of Science, Xi’an University of Architecture and Technology, Xi’an 710055, China; hebaoyi1218@163.com (B.H.); zixinhe12@163.com (Z.H.); zihanli2828@163.com (Z.L.); 2 State Key Laboratory of Cryospheric Science and Frozen Soil Engineering, Northwest Institute of Eco-Environment and Resources, Chinese Academy of Sciences, Lanzhou 730000, China; 3 Shandong Energy Group New Energy Group Co., Ltd., Jinan 250014, China; azygsc@163.com (Z.C.); zhyx82@126.com (Y.Z.); 4 Department of Mechanics and Tianjin Key Laboratory of Nonlinear Dynamics and Control, Tianjin University, Tianjin 300350, China; anshuaiwang@tju.edu.cn; 5 School of Intelligent Manufacturing and Automotive Engineering, Guangdong University of Business and Technology, Zhaoqing 526020, China; yuli10066@163.com; 6 School of Mechatronical Engineering, Beijing Institute of Technology, Beijing 100081, China; zlt960620@163.com

**Keywords:** microwave, frozen soil, dielectric properties, electromagnetic–thermal coupling, numerical simulation

## Abstract

**Highlights:**

- Water content dominates dielectric response, a frequency governed by unfrozen water, ice and matrix;
- Three-stage heating; low water content leads to faster late-stage heating; loss is negatively linked to initial content;
- EM non-uniformity leads to hot-center field, coupled model reproduces evolution.

**Abstract:**

To reveal the electromagnetic response characteristics and hydro-thermal evolution mechanism of frozen soil under microwave irradiation, we used remolded frozen soil prepared from undisturbed parent soil collected in Hegang, China, as the research object. We conducted dielectric parameter tests across the 715–1150 MHz and 2250–2650 MHz frequency bands and 1.5 kW microwave heating tests on specimens with three gravimetric water contents (15%, 20%, and 25%) paired with a coupled numerical simulation of electromagnetic field-heat transfer-moisture migration. The results show that water content is the dominant factor controlling the dielectric response of frozen soil. The dielectric loss and water content sensitivity of frozen soil in the low-frequency band (dominated by unfrozen water) are significantly higher than those in the high-frequency band (dominated by ice phase and soil matrix). Microwave-induced temperature rise exhibits a three-stage characteristic, as follows: slow temperature rise, isothermal plateau at the freezing point, and rapid temperature rise. Specimens with a lower initial water content show a higher temperature rise efficiency in the late heating stage, with a maximum rate of 1.112 °C·s^−1^ for the 15% water content specimen. Mass loss is negatively correlated with initial water content, with a maximum value of 1.8 g after 120 s of irradiation. In addition, the non-uniformity of the electromagnetic field results in a temperature field pattern characterized by a high-temperature core at the specimen center and lower temperatures at the edges. This study provides fundamental theoretical support and technical guidance for the application of microwave thawing technology in geotechnical engineering, particularly for frozen soil foundation treatment in cold regions.

## 1. Introduction

Frozen soil is a special soil type widely distributed in cold-region engineering construction, whose physical and mechanical properties are strongly governed by temperature field and moisture state. During the thawing process of frozen soil, heat input induces the coupled evolution of phase change, moisture migration and structural rearrangement, thereby affecting the deformation and stability of cold-region engineering structures including roads, tunnels and buildings [1]. With the continuous expansion of cold-region engineering construction and operation scale, higher requirements have been imposed on the efficiency, uniformity and environmental friendliness of frozen-soil thawing technologies. Traditional thawing methods (e.g., electric heating and steam heating) generally suffer from high energy consumption, non-uniform heating and long thawing duration, making it difficult for them to meet the demands of complex engineering scenarios [2]. In contrast, microwave heating features volumetric heating and selective energy absorption, enabling rapid and non-contact energy input. It exhibits promising application potential in geotechnical engineering and provides a novel technical route for efficient thawing of frozen soil [3].

The microwave heating efficiency of frozen soil is dominated by its dielectric properties. Key parameters, including relative permittivity and dielectric loss factor, determine the material’s capability of microwave energy absorption, dissipation and thermal conversion [4,5]. Existing studies generally agree that mass water content (and unfrozen water content) is the core factor controlling the dielectric response of frozen soil. The presence of unfrozen water significantly alters the polarization behavior and energy loss efficiency of frozen soil [6]. Constrained by experimental conditions, this study did not conduct direct measurement of unfrozen water content. Instead, the evolutionary characteristics of unfrozen water were inversely derived from the correlation between relative dielectric properties and temperature of the frozen soil and further verified against nuclear magnetic resonance (NMR)-measured data from similar studies. Chen et al. [7] investigated the unfrozen water content and distribution in frozen soil using NMR technology. The results show that the unfrozen water content in frozen soil is positively correlated with the initial water content and increases with a rising temperature within the range of −15 to 0 °C. This law is consistent with the physical properties of frozen soil in the Hegang region, providing reliable data support for the dielectric mechanism analysis related to unfrozen water in this paper and ensuring the rationality and scientificity of the research conclusions.

Meanwhile, the dielectric mechanism of frozen soil exhibits obvious frequency dependence: the low-frequency range is dominated by unfrozen-water-related polarization and conduction effects, while the high-frequency range is governed by contributions from the ice phase and soil skeleton, with distinct dominant mechanisms in dielectric properties [8,9]. At the typical industrial microwave frequency of 2.45 GHz, the correlation between dielectric matching characteristics and thawing efficiency has also become a research focus. Notably, frozen-soil thawing in cold-region engineering is not a pure heat conduction process, but involves the synergistic evolution of multiple processes including phase change latent heat, moisture migration and electromagnetic–temperature field coupling. However, existing studies mostly focus on the influence of single factors on thawing performance, and the systematic understanding of multi-field coupling mechanisms remains insufficient [10]. Therefore, the synergistic relationships among dielectric response, temperature rise characteristics, moisture migration and apparent morphology evolution of frozen soil under a microwave field still require further in-depth investigation.

The multi-field coupling effect of microwave-induced frozen soil thawing is essentially a two-way interaction between the dynamic evolution of dielectric properties and thermo-hydraulic processes, and the quantitative description of this interaction mechanism in existing studies still has obvious shortcomings. The dynamic change in unfrozen water content during the phase transition process will continuously alter the dielectric response characteristics of frozen soil, which in turn changes the spatial distribution of microwave energy deposition and forms a non-linear positive feedback loop with the temperature field evolution [11]. Laboratory tests have confirmed that microwave heating can achieve rapid thawing of artificial frozen soil, but the non-uniformity of temperature field caused by the difference in dielectric properties of each phase component will lead to local overheating and structural deterioration of soil. Mesoscopic test results further reveal that the thawing sequence of ice crystals in frozen soil under microwave irradiation is controlled by the local dielectric loss level, and the evolution of mesoscopic pore structure will in turn affect the macroscopic thawing efficiency [12]. Most existing hydro-thermal coupling models for frozen soil focus on the freeze–thaw process under natural temperature boundary, and rarely consider the active energy input of microwave and the dynamic change in dielectric parameters with temperature and water content [13]. The fully coupled electromagnetic–thermal–mass transfer model established for microwave heating of porous media provides a theoretical framework for this study, but the model parameters need to be corrected according to the phase change characteristics of frozen soil to ensure the accuracy of the numerical simulation [14].

In recent years, systematic studies have been conducted on the dielectric response and microwave heating mechanism of frozen soil. It is widely recognized that the coupled effects of gravimetric water content, temperature, and frequency significantly influence the wave-absorbing performance and heating rate of frozen soil. Jia et al. [15] investigated clayey silt using temperature measurement, nuclear magnetic resonance, and unconfined compressive strength tests, revealing the thermal-softening process and microstructural evolution of frozen soil under microwave irradiation. They identified unfrozen water as the key controlling factor in thawing and softening. For multi-field coupling description, Wei et al. [16] established a fully coupled electromagnetic–thermal–mass transfer model for microwave heating, providing a theoretical foundation for process simulation of frozen soil thawing. Hamid et al. [17] proposed a novel microwave power radiator structure, offering device-level design support for engineering applications in frozen soil thawing and similar outdoor scenarios.

Despite these advances, existing research still exhibits limitations, as follows: first, the dominant factors and energy loss mechanisms of dielectric properties in different frequency bands-especially the low-frequency range of 715–1150 MHz and high-frequency range of 2250–2650 MHz-lack targeted experimental verification and unified understanding [18]. Second, quantitative coupling between the staged heating characteristics of microwave irradiation and moisture migration/mass loss remains insufficient, the migration mechanism under the combined action of gravimetric water content and temperature gradient requires further refinement [19]. Third, parameter selection in multi-field coupling numerical models still relies heavily on empirical assumptions, and matching with experimental boundaries and material properties needs improvement. Fourth, no correlation has been established between surface morphological evolution and internal physical changes, hindering the provision of direct evidence for engineering monitoring and quality evaluation [20].

The above limitations directly restrict the transformation of microwave frozen-soil thawing technology from laboratory research to engineering application, and it is urgent to establish a complete research system including dielectric property test, multi-field coupling simulation, and engineering parameter optimization. The stability of engineering structures in permafrost regions is highly sensitive to the thawing rate and uniformity of frozen soil, and traditional thawing methods can no longer meet the construction requirements of high-grade highways, railways and long-distance tunnels in complex cold regions [21]. The existing dielectric property test results of frozen soil are mostly obtained under specific frequency and temperature conditions, and the lack of systematic test data in the full-frequency band of industrial microwave makes it difficult to provide direct guidance for the selection of engineering-equipment parameters [22]. Field tests of microwave thawing of artificial frozen soil in tunnel engineering have verified the feasibility of this technology, but the quantitative matching relationship between microwave power, irradiation duration, soil water content and thawing depth has not been clarified [23]. In the context of global warming, the thaw settlement of frozen soil foundation has become the core problem restricting the long-term stability of cold region engineering, and efficient and controllable thawing technology is an important prerequisite for emergency repair and maintenance of frozen soil engineering [24]. The seasonal frozen soil regions in China are widely distributed, and the soil types, water content and environmental conditions of engineering projects in different regions vary greatly, which puts forward higher requirements for the universality and adaptability of microwave thawing technology [25].

In addition, existing studies have explored microwave thawing of frozen soil from multiple aspects: Research on dielectric loss mechanisms provides theoretical support for improving heating efficiency [26], experimental optimization of process parameters offers data references for engineering applications [27], the influence of temperature–frequency coupling on dielectric properties further refines the dielectric response theory [28]. In numerical simulation, review studies on multi-physics coupling summarize the current status and development trends [29], experimental and numerical analyses of thermal response enable detailed characterization of temperature evolution during thawing [30,31]. Meanwhile, studies linking surface morphology and mechanical performance provide new perspectives for engineering quality assessment [32], the development and validation of thermo–hydro–mechanical coupling models extend the application of multi-field theory in frozen soil engineering [33], investigations into the effects of frequency and gravimetric water content on thawing performance offer direct guidance for parameter selection and optimization. For engineering practice, parametric optimization tests support field application [34], reviews of subgrade thawing technology for cold-region highways clarify the application potential of microwave heating in road engineering [35]. Numerical analysis of temperature and moisture distribution enables high-fidelity simulation of thawing processes, comparative studies on dielectric properties across frequencies deepen understanding of frequency-dominant mechanisms, experimental work on thawing efficiency at different water contents provides data for engineering parameter design, and the development and validation of multi-field coupling models enhance the reliability of numerical simulation [36].

This study poses a core scientific question: How do water content, frequency, and temperature jointly regulate the dielectric polarization and energy dissipation mechanisms of frozen soil, and what is the intrinsic coupling relationship between dielectric response, staged temperature rise, moisture migration, and surface morphological evolution during microwave thawing? To systematically answer this question, we use remolded frozen soil samples prepared from undisturbed parent soil collected in Hegang, Heilongjiang, Northeast China, as the research object. We test samples with different initial gravimetric water contents on a self-built 2.45 GHz microwave heating platform, and conduct systematic experiments paired with thermal imaging and radio-frequency dielectric measurements to characterize the relative permittivity of frozen soil, monitor temperature evolution during microwave heating, and quantify moisture loss and temperature field distribution. We further establish a multi-physics coupling numerical model integrating electromagnetic field, heat transfer, and moisture migration to interpret and verify the internal relationships among temperature field heterogeneity, moisture migration, and dielectric response under microwave irradiation. This work clarifies the effects of gravimetric water content, frequency, and temperature on the dielectric loss mechanism and wave-absorbing efficiency of frozen soil, reveals the synergistic mechanism between staged thawing behavior and multi-physics coupling response, and provides theoretical and technical support for parameter optimization and engineering application of microwave thawing technology in cold-region engineering.

The objective of this work is to reveal the dielectric characteristics and electromagnetic–thermo-hydraulic coupling mechanism of frozen soil under microwave irradiation. The paper is organized as follows: Section 2 details the sample preparation and experimental procedures, including dielectric parameter measurement, microwave heating test, and temperature–mass loss monitoring. Section 3 reports the dielectric properties of frozen soil and the effects of water content, frequency, and temperature. Section 4 presents the staged temperature rise, moisture migration, and surface morphology evolution during microwave heating. Section 5 establishes a three-dimensional electromagnetic–thermal–hydraulic coupling model and analyzes the non-uniform distribution of temperature, electromagnetic field, and water concentration. Section 6 summarizes the key findings and provides engineering implications for microwave thawing of frozen soil.

## 2. Sample Preparation and Experimental Program

### 2.1. Sample Preparation

The soil used in this study was collected from Hegang, Heilongjiang Province, Northeast China, and is classified as typical seasonally frozen silty clay. Its particle-size distribution, mineral composition, and basic physical properties are representative of the frozen-soil engineering characteristics in the study area. Considering that undisturbed frozen soil sampling is prone to structural disturbance, heterogeneous pore distribution, and difficulty in precisely controlling moisture gradients, remolded samples were adopted in this study to ensure single-variable control, data repeatability, and intergroup comparability. The parent soil was obtained by crushing and sieving undisturbed frozen soil from Hegang, which preserved the particle-size distribution and mineral background to the greatest extent while eliminating interference from natural heterogeneity of undisturbed samples. To guarantee repeatability and comparability, all specimens were remolded from the same parent soil and prepared in accordance withthe Standard for Geotechnical Testing Method (GB/T 50123-2019 China Planning Press: Beijing, China, 2019) [37]. Cubic specimens with dimensions of 100 mm × 100 mm × 100 mm were used, balancing test operability, measurement accuracy, and representativeness of engineering loading conditions. Basic physical index tests of the parent soil showed a liquid limit of 30.6%, plastic limit of 18.4%, and saturated water content of 46.5%. For sample preparation, the parent soil was first dried in an oven for 12 h, then crushed and passed through a 2 mm sieve. The sieved soil was divided into three groups and mixed with water to target gravimetric water contents of 15%, 20%, and 25%, respectively. Each mixture was sealed and stored for 24 h to achieve a uniform moisture distribution. The soil was then compacted layer-by-layer into cubic molds. Three parallel specimens were prepared for each water content, yielding a total of 9 frozen samples. After molding, specimens were wrapped with plastic film and frozen in a temperature-controlled refrigerator at −15 °C for 48 h to form frozen soil samples. The measured wet density of all prepared specimens was 1.73 g·cm^−3^. Uniform wet density was strictly controlled in this test to simulate the natural wet density distribution of in situ frozen soil in cold regions. Under natural deposition and compaction, frozen soil within the same field region generally exhibits consistent wet density, whereas water content varies significantly due to moisture migration and freeze–thaw cycles. Therefore, the sample preparation scheme with identical wet density is more consistent under actual engineering conditions.

### 2.2. Experimental Program

**Figure 1:**
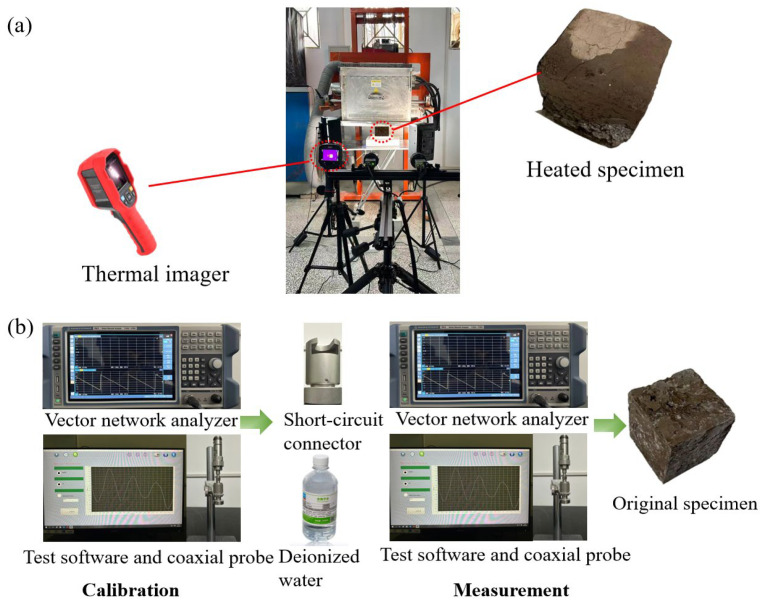
Test apparatus and experimental procedure: (**a**) microwave heating test procedure; (**b**) relative dielectric constant measurement procedure.

The experimental setup in this study is shown in Figure 1, which includes microwave heating and relative permittivity measurement procedures. The instruments consisted of a self-built open microwave heating system, a thermal imager, and a vector network analyzer (VNA). As shown in Figure 1a for the microwave heating procedure, a custom open microwave heating device (2.45 GHz) was adopted, equipped with a multi-channel air-cooled microwave power supply and magnetron, with a maximum output power of 12 kW. It enables continuous irradiation over an area of approximately 100 mm × 370 mm for more than 10 min and is fitted with microwave leakage protection and high-temperature burst protection for specimens. Temperature field monitoring was performed using a UNI-T RM600F (UNI-T, Dongguan, China) thermal imager (640 × 512 pixels, measurement range −20 to 160 °C, accuracy ± 2 °C). As illustrated in Figure 1b, dielectric property measurements of frozen soil were conducted using a Rohde & Schwarz ZNL3 VNA (Rohde & Schwarz GmbH & Co. KG, Munich, Germany) via the coaxial method.

(1) Experimental Program for Relative Permittivity Measurement

Procedure: Dielectric property testing of frozen soil was performed using the VNA, with system calibration completed via the coaxial single-end open-ended method, supported by supporting software, a coaxial adapter, and a short-circuit connector. After calibration, the specimen was placed on the workbench, and the flange probe was pressed firmly against a relatively flat surface of the specimen for measurement. The dielectric response of frozen soil is governed by particle-size distribution, water content, test frequency, ice phase, and unfrozen water content.

(2) Experimental Program for Temperature Measurement

Procedure: The test environment was controlled at a constant temperature of 20 ± 1 °C. Frozen soil specimens with 15%, 20%, and 25% water contents were placed under the waveguide of the custom open microwave heating device, with thermal insulation panels installed around for protection. Boundary heat loss was corrected using a heat conduction model, the calculated influence of heat dissipation upon temperature rise was less than 5% and thus negligible. The microwave equipment cooling system was activated, and the power was set to 1.5 kW for 120 s of heating. Thermal images were captured every 10 s during the 0–120 s period. The thermal imager accuracy of ±2 °C satisfies the requirements for measuring the macroscopic temperature field and temperature rise trend in this study. All test data were verified by three repeated measurements and error calibration to ensure reliability. Furthermore, internal temperature measurement was not feasible due to the physical characteristics of frozen soil specimens. A validated electromagnetic–thermal–moisture migration coupled numerical model was used to simulate the internal temperature evolution of 15% water content frozen soil. By comparison with surface thermal imaging data, the internal-surface temperature correlation was established, which numerically verified the non-uniform heating characteristic of preferential surface warming and rapid energy attenuation with depth.

(3) Experimental Program for Heating Characteristics and Moisture Loss

Moisture in frozen soil exhibits high dielectric loss, leading to strong wave absorption and thermal conversion under microwave irradiation. As a key parameter affecting microwave heating efficiency, the mechanism of water content remains to be clarified. Therefore, comparative microwave heating tests were conducted on frozen soil with different water contents. A relatively low power of 1.5 kW was selected to reduce coupling interference from other factors under high power, ensuring the specificity and reliability of test results. Surface temperature evolution and specimen mass change were recorded continuously during testing: temperature rise was recorded up to 120 s, and mass variation was also measured at 120 s. After each heating cycle, specimen mass before and after heating was weighed using an electronic balance (accuracy: 0.1 g) to characterize the temperature rise pattern and moisture escape (Moisture Loss).

## 3. Experimental Results of Relative Permittivity of Frozen Soil

### 3.1. Analysis of Dielectric Parameter Characteristics of Frozen Soil

To quantitatively evaluate the energy absorption and conversion capability of frozen soil in a microwave field, the complex relative permittivity is generally adopted to characterize the overall response of the medium to the electromagnetic field [38], as shown in Equations (1)–(3).

(1) $$\varepsilon =\varepsilon_{0}\varepsilon_{r}=\varepsilon_{0}\left(\varepsilon^{\prime }-j\varepsilon^{\prime \prime }\right),$$

(2) $$W=\frac{1}{2}\varepsilon_{0}\varepsilon^{\prime }E^{*}\cdot E,$$

(3) $$W^{\prime }=\frac{1}{2}\varepsilon_{0}\varepsilon^{\prime \prime }E^{*}\cdot E,$$

where $\varepsilon_{r}$ is the complex relative permittivity (dimensionless), $\varepsilon^{\prime }$ is the real part of relative permittivity (dimensionless), $\varepsilon^{\prime \prime }$ is the imaginary part of relative permittivity (dimensionless), j is the imaginary unit (dimensionless), ε is the complex absolute permittivity ($F\cdot m^{-1}$), $\varepsilon_{0}$ is the vacuum permittivity ($F\cdot m^{-1}$).

The complex relative permittivity consists of the following two components: the real part of relative permittivity (denoted as ε′) and the imaginary part (denoted as ε″). Among them, ε′ reflects the ability of the medium to polarize and store electromagnetic energy under an alternating electric field, ε″, also known as the dielectric loss factor, represents the capacity of the medium to dissipate electromagnetic energy into thermal energy and other forms. To further quantify the relative loss per unit cycle, the dielectric loss tangent (tan δ) is introduced, defined by Equation (4): tan δ = ε″·ε′^−1^. Physically, tan δ represents the ratio of energy dissipated to energy stored within one cycle, and serves as a key indicator for evaluating the microwave heating efficiency of materials.

(4) $$\tan\delta =\frac{\varepsilon^{\prime \prime }}{\varepsilon^{\prime }},$$

Using the aforementioned automatic dielectric measurement system, relative permittivity tests were performed on frozen soil specimens with water contents of 15%, 20%, and 25% (labeled S15, S20, S25, respectively, where the number indicates the water content percentage). Each specimen was tested five times, and the average value was taken as the measured dielectric parameter. The relative standard deviation (RSD) of parallel specimens in each group was less than 5%, indicating acceptable measurement error. The test covered a low-frequency band of 715–1115 MHz and a high-frequency band of 2250–2650 MHz. These ranges were selected because they include typical industrial microwave frequencies (915 MHz, 2450 MHz), ensuring relevance to engineering applications, while avoiding interference bands to guarantee data reliability. Constrained by experimental conditions, direct measurement of unfrozen water content was not performed in this study. Instead, the evolution of unfrozen water was inversely derived from the correlation between relative dielectric properties and temperature. This interpretation was supported by measured data from similar studies on unfrozen water content in frozen soil [39]: under the same initial water content, unfrozen water content decreases with decreasing temperature in cold frozen soil, moreover, a higher initial water content leads to a more pronounced reduction in unfrozen water content at low temperatures, eventually approaching a stable value. This evidence ensures the rationality and reliability of the dielectric mechanism analysis related to unfrozen water.

**Figure 2:**
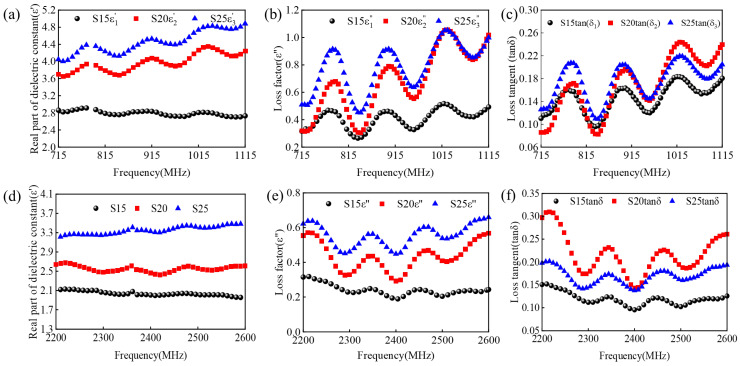
Test results of dielectric properties of frozen soil materials: (**a**) Real part of low-frequency relative permittivity, (**b**) Imaginary part of low-frequency relative permittivity, (**c**) Low-frequency loss tangent, (**d**) Real part of high-frequency relative permittivity, (**e**) Imaginary part of high-frequency relative permittivity, (**f**) High-frequency loss tangent.

The low-frequency test range was 715–1115 MHz (Figure 2a–c). The results show that ε′ differs significantly among the three groups and increases monotonically with water content (S25 > S20 > S15), indicating that higher water content substantially enhances the polarization and energy storage capacity of frozen soil. Meanwhile, ε″ and tan δ rise slowly with increasing frequency and exhibit noticeable fluctuations within this band. The primary cause is the strong electromagnetic response of unfrozen water inside frozen soil: the rotational polarization of unfrozen water molecules under an alternating electric field is characterized by a relaxation time, and the lag of dipole reorientation with changing frequency leads to fluctuations in dielectric parameters. A higher water content strengthens the contribution of unfrozen water and amplifies such fluctuations.

The high-frequency test range was 2250–2650 MHz (Figure 2d–f). The overall trend is consistent with the low-frequency band: ε′ still increases with water content, but the curves become smoother and frequency-dependent fluctuations are weakened. ε″ and tan δ also increase slightly with frequency, but with reduced amplitude, showing a more stable frequency response. A comparison between low and high frequencies reveals that in the low-frequency band, the dielectric response is dominated by unfrozen water, and the loss level and fluctuation are more sensitive to water content. In the high-frequency band, contributions from the soil matrix and ice phase become more significant, leading to a more stable dielectric response and a relatively weaker influence of water content. These results reveal the key controlling factors of wave absorption and dissipation in frozen soil from an electromagnetic response perspective, providing a basis for subsequent parameter selection and energy regulation in microwave thawing.

### 3.2. Effects of Frequency and Temperature

The commonly used industrial microwave frequencies are 915 MHz and 2450 MHz. Investigating these two frequencies is practically meaningful because 915 MHz microwave exhibits strong penetration, suitable for thick-layer frozen soil thawing, 2450 MHz features high power density and low equipment cost, suitable for small-scale thawing scenarios. Clarifying the dielectric differences at these two frequencies provides a direct basis for frequency selection and parameter matching in engineering. Understanding the dielectric behavior at these frequencies is therefore critical for frozen soil microwave thawing. In this study, dielectric properties of the raw soil, ice, and water were tested, each material parameter was averaged over five measurements. Averaged values were also adopted for frozen soil specimens with different water contents. According to the real part of relative permittivity, materials are classified as polar (>3.6), weakly polar (2.8–3.6), and nonpolar (<2.8).

As shown in Table 1, at 915 MHz, water and soil are strongly polar, while ice is weakly polar. The real part of relative permittivity of frozen soil increases with water content, and the material polarity shifts from weakly polar to strongly polar. This indicates that unfrozen water significantly enhances the dielectric response and energy dissipation of frozen soil.

As listed in Table 1, at 2450 MHz, water remains strongly polar, whereas soil and ice are generally weakly polar. The real part of relative permittivity of frozen soil still increases with water content but is lower than at 915 MHz, revealing obvious frequency sensitivity. Under high frequency, the relative contributions of the soil matrix and ice phase to the dielectric response are enhanced.

**Figure 3:**
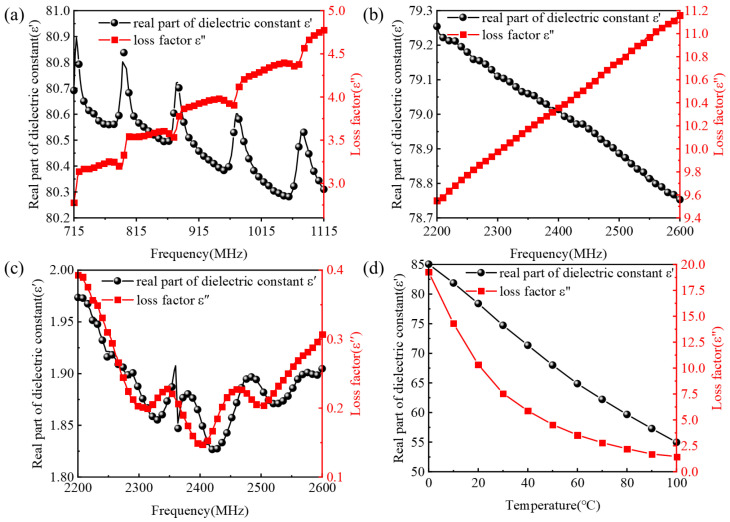
Frequency- and temperature-dependent relative permittivity of water and ice measured via the coaxial probe method: (**a**) variation in the real part of relative permittivity of water with frequency in the 715–1150 MHz low-frequency band; (**b**) variation in the real part of relative permittivity of water with frequency in the 2250–2650 MHz high-frequency band; (**c**) frequency-dependent complex relative permittivity of ice; (**d**) temperature-dependent complex relative permittivity of water at the industrial microwave frequency of 2450 MHz.

Figure 3a,b present the frequency-dependent dielectric parameters of water at room temperature measured by the coaxial method: the real part decreases with increasing frequency, while the loss factor and loss tangent increase, indicating that higher frequency reduces energy storage capacity but strengthens dissipation. Figure 3c shows that the real permittivity ε’ and loss factor ε’’ of ice first decrease and then fluctuate upward, related to interfacial polarization at ice–gas boundaries and dipole relaxation from lattice defects. Figure 3d shows that the complex permittivity of water decreases rapidly with increasing temperature, the real part declines approximately linearly, and the loss factor drops sharply then slows at higher temperatures, implying that heating weakens the microwave absorption capacity of water. Thus, the energy coupling efficiency of frozen soil microwave thawing is highly sensitive to the combination of water content, frequency, and temperature stage. Higher water content strengthens dielectric response, but dielectric parameters decay with temperature rise. Frequency selection and heating duration must be coordinated in process design. Furthermore, the dielectric characteristics near the freezing point (phase-change region) and the unfrozen-water-dominated range should be emphasized to improve energy absorption efficiency in the early thawing stage while ensuring heating uniformity.

## 4. Experimental Results of Frozen Soil Under Microwave Irradiation

### 4.1. Heating Process and Stage Characteristics

**Figure 4:**
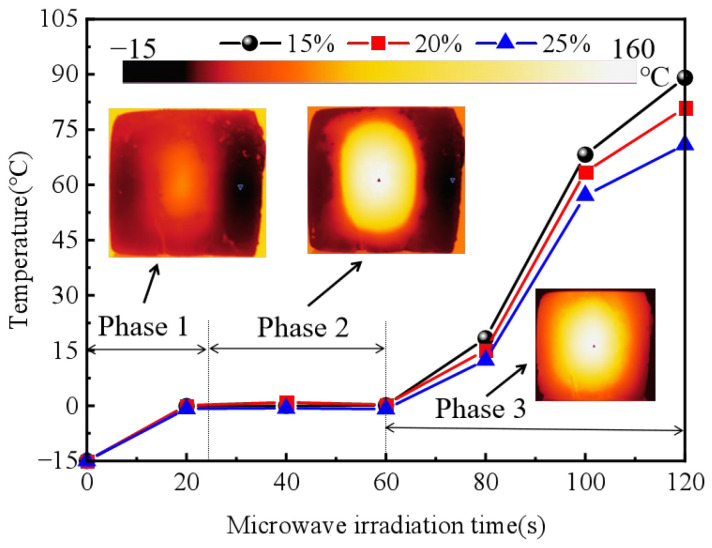
Temperature rise characteristics of frozen soil with different moisture contents.

Temperature rise curves of frozen soil with different water contents (15%, 20%, 25%) are presented in Figure 4. Based on curve morphology, the heating process can be divided into the following three stages: (1) Slow heating stage—surface temperature rises gradually at a rate of approximately 0.375 °C∙s^−1^, with small temperature differences among groups; (2) isothermal plateau near freezing point—temperature remains nearly constant at approximately 0 °C, indicating that input energy is mainly consumed by latent heat of phase change; (3) rapid heating stage—after the plateau, surface temperature increases sharply. Samples with lower water content exhibit faster heating in the later period (representative rates are about 1.112, 1.007, and 0.897 °C∙s^−1^ for 15%, 20%, and 25% samples, respectively). When temperature exceeds approximately 60 °C, the heating rate slows down in all groups.

### 4.2. Moisture Loss Characteristics

**Figure 5:**
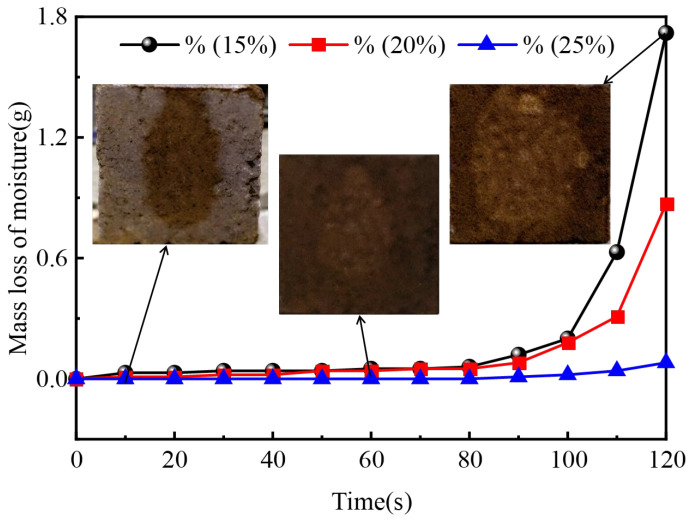
The quality loss curve during microwave thawing process.

Moisture Loss curves of frozen soil under 1.5 kW microwave heating are shown in Figure 5. Mass loss mainly results from water vaporization and escape under microwave heating. In the early thawing stage (0–80 s), mass loss remains near zero-gram with no obvious moisture escape. In the later stage, the low-water-content group shows earlier mass loss, followed by the medium group, while the high-water-content group remains relatively stable. At 120 s, mass loss reaches approximately 1.8 g and 0.8 g for the low and medium groups, respectively. An overall negative correlation between moisture loss and initial water content is observed.

Comparison between temperature rise and mass loss curves shows that moisture escape occurs even when internal and surface temperatures are below the atmospheric boiling point. This phenomenon can be explained by the coupled mechanism, as follows: temperature gradient→vapor pressure gradient→moisture migration. Microwave selective absorption and volumetric heating cause preferential heating in high-moisture regions, with internal temperature rising faster than the surface, forming an inward-to-outward temperature gradient. Local vaporization within pores generates a vapor pressure gradient of 10–30 kPa, consistent with the critical migration pressure (≥10 kPa) measured by Zhang C. Y. [32] for vapor diffusion in frozen soil. This gradient is sufficient to drive moisture migration and escape. The negative correlation between mass loss and initial water content further confirms that more uniform pore moisture distribution in high-water-content samples delays vapor pressure buildup and reduces moisture escape efficiency.

### 4.3. Evolution Characteristics of Frozen Soil Surface Morphology Under Microwave Irradiation

**Figure 6:**
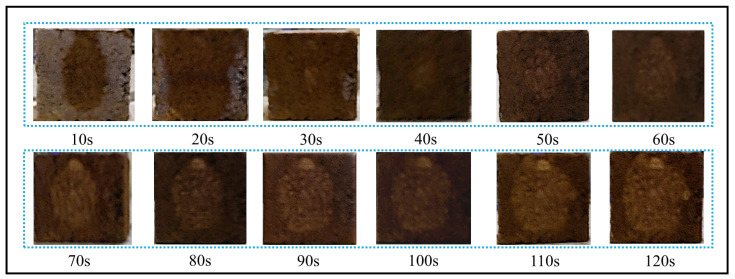
The apparent morphology of the microwave thawing process. The blue dashed boxes are visual guides that frame each time-step snapshot for clarity.

As shown in Figure 6, the surface morphology of frozen soil exhibits distinct staged evolution during microwave irradiation: At the initial thawing stage, the specimen surface is generally flat with only slight local melting traces. As thawing proceeds, ice crystals gradually ablate, forming obvious undulating textures and increasing surface roughness. At the late thawing stage, surface textures are fully developed and distributed uniformly. The above morphological evolution is consistent with the selective absorption-volumetric heating mechanism of microwave heating. As a high-loss component, unfrozen water preferentially absorbs microwave energy and heats up first, forming local melting and heat concentration zones inside the specimen. Heat then transfers to surrounding ice and soil particles, promoting the expansion of the melting zone. This drives the surface to evolve gradually from local melting to global thawing textures.

## 5. Electromagnetic–Heat Coupled Numerical Model for Frozen Soil Under Microwave Irradiation

### 5.1. Finite Element Model

**Figure 7:**
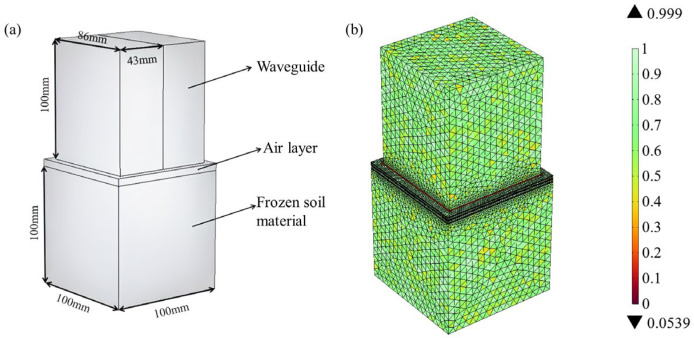
Model establishment: (**a**) geometric model; (**b**) mesh generation.

In the numerical modeling of an open microwave heating device, the settings for frozen soil material parameters, electromagnetic field parameters, and control equations (PDEs) remain consistent with those of the resonant cavity microwave heating model. The primary differences lie in the form of the reflective structure and the number and arrangement of microwave ports. To more realistically represent the spatial distribution characteristics of the electromagnetic and temperature fields within frozen soil under open heating conditions, this study establishes a three-dimensional finite element model for the thawing of frozen soil using an open microwave heating system. The model adopts sample dimensions and operating conditions that match the experiments. The frozen soil sample in the model is a cube measuring 100 mm × 100 mm × 100 mm. Microwave irradiation is introduced through a top port, with two rectangular waveguide ports set on the top surface. Each waveguide has dimensions of 86 mm × 43 mm × 100 mm, and the bottom of the waveguide port is positioned 3 mm above the top surface of the frozen soil. The microwave operating frequency is set at 2.45 GHz, with an input power of 1.5 kW. The schematic of the geometric model and mesh division is shown in Figure 7.

The Maxwell equations elucidate the physical relationship between electricity and magnetism, comprising four fundamental equations that comprehensively describe the interaction and variation laws of electric and magnetic fields. Maxwell’s theory not only explains the connection between these two fundamental forces in the universe but also predicts the existence of electromagnetic waves, emphasizing the interdependence and conversion relationship between electric and magnetic fields. These equations describe how electric and magnetic fields interact and change in space and time. For electromagnetic waves in free space, the Maxwell equations can be simplified to the following forms:

(5) $$\begin{array}{l} \nabla \cdot D=\rho \\ \nabla \cdot B=0\\ \nabla \times E=\frac{\partial B}{\partial t}\\ \nabla \times H=J+\frac{\partial D}{\partial t} \end{array},$$

where D represents the electric displacement field (C/m^2^), ρ represents the charge density (C/m^3^), B represents the magnetic flux density (Wb/m^2^), E represents the electric field intensity (V/m), H represents the magnetic field intensity (A/m), and J represents the surface current density (A/m^2^).

For linear, isotropic materials, the constitutive relationships between the electric displacement field and the electric field intensity, as well as between the magnetic flux density and the magnetic field intensity, it can be expressed as follows:

(6) $$\begin{array}{l} D=\varepsilon_{0}\varepsilon_{r}E\\ B=\mu_{0}\mu_{r}H \end{array},$$

where $\varepsilon_{0}$ is the vacuum permittivity, $\varepsilon_{r}$ is the relative permittivity of the material, $\mu_{0}$ is the vacuum permeability, $\mu_{r}$ is the relative permeability of the material.

When solving the electromagnetic field model under harmonic excitation, frequency domain analysis is typically used, allowing the relationship between the electric field and time to be transformed into the following form:

(7) $$E=E_{0}e^{j\omega t},$$

The relative permittivity of the material is expressed as follows:

(8) $$\varepsilon_{r}=\varepsilon^{\prime }-j\varepsilon^{\prime \prime },$$

where $\varepsilon^{\prime }$ represents the dielectric constant, measuring the degree of polarization of the material under an electric field, $\varepsilon^{\prime \prime }$ is the loss factor of the material, indicating the absorption and loss of electric field energy by the material.

Thus, the dielectric loss of the material is solely related to the loss factor. The resistive power loss density is expressed as follows:

(9) Qrh=Re(12jωε0εrE⋅E)=12ωε0ε″E⋅E,

where Re is the only real part taken.

For frozen soil, magnetic induction heating is essentially not involved, so there is no magnetic loss. Therefore, the electromagnetic heating generated by the microwave is as follows:

(10) $$Q_{e}=Q_{rh},$$

For the frozen soil portion, it is considered as a multiphase porous medium, and its thermophysical properties change according to the volumetric proportion of each phase. The energy balance equation and the calculation formulas for effective thermodynamic parameters are as follows:

(11) $$\rho C_{p}\frac{\partial T}{\partial t}+\sum_{i}\left(u_{i}\nabla \left(C_{p,i}T\right)\right)=\nabla (K_{i}\nabla T)-\lambda \dot{I}+Q_{m},$$

where ρ represents density (kg/m^3^), $C_{p}$ represents specific heat capacity (J/kg·K), $K_{i}$ represents thermal conductivity (W/m·K), λ = 334 (J/kg) denotes the latent heat of phase change. $C_{ps}$, $C_{pi}$ and $C_{pw}$ represent the specific heat capacities of the solid matrix, ice and liquid water (J/kg·K), respectively. $K_{s}$, $K_{i}$ and $K_{w}$ represent the thermal conductivities of the solid matrix, ice and liquid water (W/m·K), respectively.

(12) $$u=-\frac{k}{\mu }\nabla \rho ,$$

where u is the Darcy velocity vector, measured in m·s^−1^. p is the fluid pressure, measured in Pa; μ is the resin viscosity, measured in Pa·s. K is the permeability tensor, measured in m^2^, representing the resistance characteristics of the fiber preform to fluid flow.

(13) $$I=\left\{\begin{array}{l} e^{{\left(\frac{T-273.15}{W}\right)}^{2}},T<273.15\\ 1,T\geq 273.15 \end{array}\right.,$$

In this expression, I the value range of is between 0 and 1, representing the degree of phase change. When I=0, it is ice, when I=1, it is water.

This model is used to simulate the distribution of electromagnetic fields, electromagnetic power loss, temperature fields, and the pathways and rates of water migration in frozen soil under microwave heating. The frozen soil material is taken from the original frozen soil used in the experiments, and the matrix is considered a non-deformable medium in the calculations.

Other parameters in the microwave thawing model of frozen soil are listed in Table 2.

### 5.2. Temperature Field Distribution Characteristics

During microwave heating, the spatial distribution of the temperature field in frozen soil is jointly controlled by the material’s dielectric properties and the electromagnetic field distribution. The open system used in this study belongs to a single-mode heating form. Although its electromagnetic field distribution is relatively regular, due to the influence of waveguide structure, boundary reflections, and coupling conditions, there are still significant spatial differences in the internal electric field intensity and power loss density of the sample. This leads to a non-uniform temperature field, which may further affect water migration and thawing efficiency.

To obtain the surface temperature field distribution characteristics of the frozen soil after microwave irradiation, a UNI-T RM600F thermal imaging camera (640 × 512, −20 ~ 150 °C, ±2 °C) was used for continuous observation of the sample surface. The microwave heating duration was set from 0 to 120 s, with thermal images collected at 10 s intervals. Taking a sample with 15% water content as an example, the evolution of the temperature field on the front and side surfaces over time is presented and compared with numerical simulation results under the same operating conditions.

(1) Temperature Field Distribution and Evolution on the Irradiated Surface

**Figure 8:**
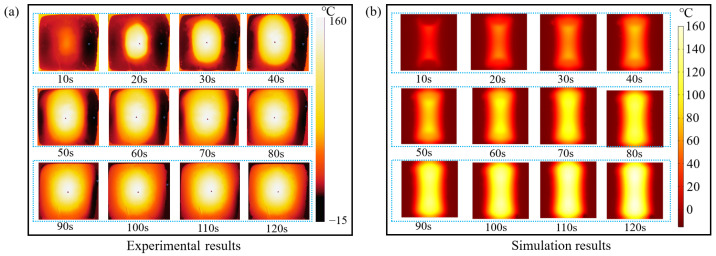
Frontal temperature distribution map: (**a**) test results; (**b**) simulation results. The blue dashed boxes are visual guides that frame each time-step snapshot for clarity.

The front thermal image shows that the temperature field exhibits a clear non-uniform distribution (as shown in Figure 8), accompanied by an evolution process of “hot spot—expansion—convergence”, as shown in Figure 8: multiple discrete hot spots appear in the early stage of heating, subsequently, the hot spots rapidly expand and undergo connection and merging, forming a continuous high-temperature zone, in the later stage of heating, the central region gradually evolves into a stable high-temperature core zone, while the edge temperature remains relatively low, with a significant temperature difference between the center and the edge. This phenomenon indicates that the spatial deposition of microwave energy is non-uniform, and the high-field-strength region has a stronger ability to absorb waves and generate heat. Mechanistically, electromagnetic field non-uniformity first leads to local differences in power loss density, during the thawing progression, the increase in unfrozen water content enhances dielectric loss, making high-field-strength regions prone to form a positive feedback loop of “energy deposition-temperature rise-melting-enhanced loss”, thereby intensifying hot spots and exacerbating temperature field non-uniformity.

**Figure 9:**
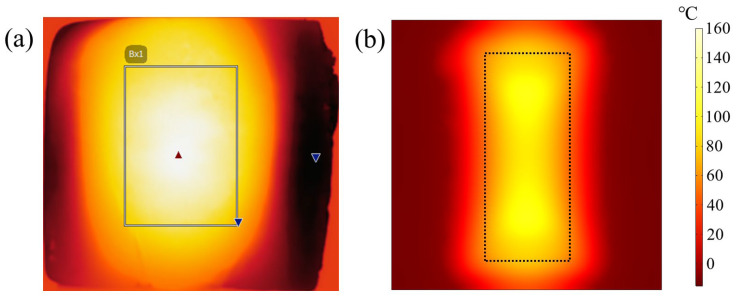
Front temperature distribution after 60 s of heating: (**a**) experimental results; (**b**) simulation results. The box indicates that the temperature is above 80℃.The red triangle represents the highest temperature, while the blue triangle represents the lowest temperature.

To quantitatively verify the model accuracy, five characteristic measurement points (one center and four corners) were selected on the front of the sample. The characteristic measurement points at four moments (30 s, 60 s, 90 s, 120 s) were extracted for error analysis (as shown in Table 3): the average relative error (MRE) between simulation and experiment is 4.72%, and the root mean square error (RMSE) is 3.85 °C, both meeting the accuracy requirements for geotechnical multi-physics coupling simulations (MRE < 5%). The simulation and thermal image results are generally consistent in the location, shape, and temporal evolution trend of the high-temperature zone, indicating that the constructed model can effectively represent the main distribution patterns of the temperature field in frozen soil under open microwave heating. As shown in Figure 9, there is a slight difference in the size of the high-temperature zone (temperature > 80 °C) between the experiment and simulation. The high-temperature zone area in the experiment is about 32 cm^2^, while the simulation result is about 29 cm^2^. This discrepancy is caused by the simplification of temperature dependence of thermal physical parameters in the model and the idealization of boundary heat dissipation conditions.

(2) Temperature Field Distribution and Evolution on the Side Surface

**Figure 10:**
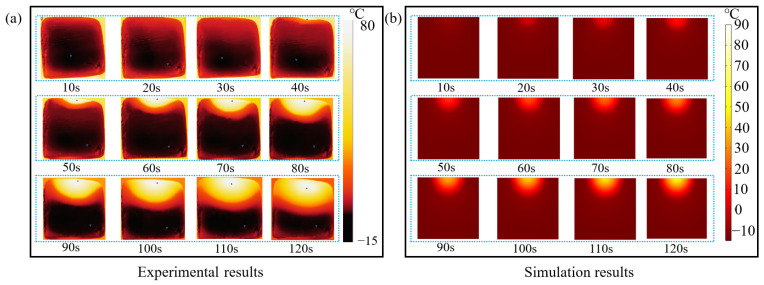
Side view temperature distribution map: (**a**) test results; (**b**) simulation results. The blue dashed boxes are visual guides that frame each time-step snapshot for clarity.

The side thermal image indicates that the temperature field is not formed by unidirectional conduction from the surface to the interior, but rather reflects the “volumetric heating” characteristic of microwaves, as shown in Figure 10: in the early stage of heating, multiple hot spots can already appear inside the sample, in the mid-stage, hot spots expand around and interconnect, while heat diffuses to low-temperature zones through conduction, in the later stage, the central region continues to accumulate energy, forming a stable high-temperature core zone, while the edge region lags in temperature rise, and the internal temperature gradient becomes more pronounced. The comparison between experiment and simulation shows that both maintain consistency in the spatial distribution and evolution trend of the high-temperature zone, further verifying the model’s ability to represent temperature field non-uniformity and hot spot evolution. Local deviations may also stem from simplified handling of material parameters and boundary conditions.

(3) Internal Temperature Field Distribution and Evolution

**Figure 11:**
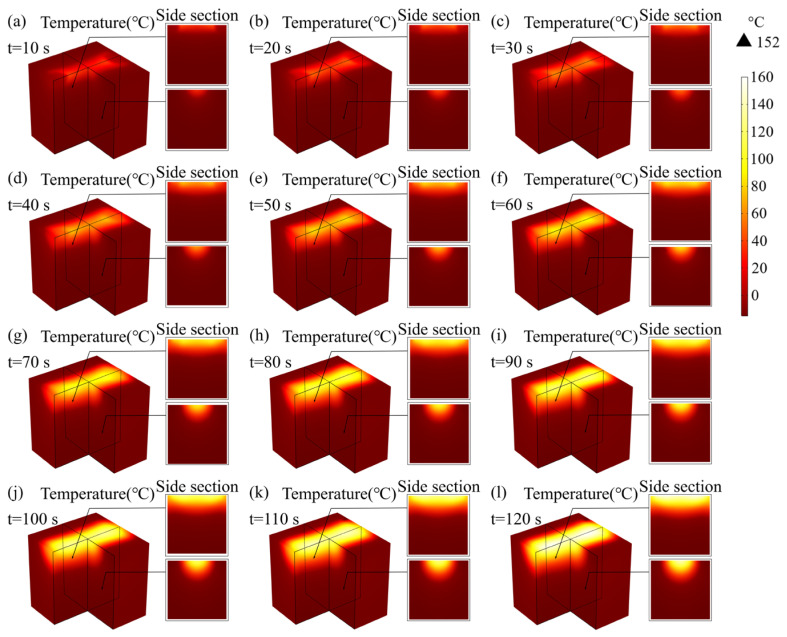
Temperature distribution within the frozen soil over a period of 10 to 120 s (**a**) 10 s, (**b**) 20 s, (**c**) 30 s, (**d**) 40 s, (**e**) 50 s, (**f**) 60 s, (**g**) 70 s, (**h**) 80 s, (**i**) 90 s, (**j**) 100 s, (**k**) 110 s, (**l**) 120 s.

Figure 11 demonstrates the evolution pattern of the internal temperature field of frozen soil during microwave heating. Overall, the high-temperature region preferentially appears in the upper region of the cube, and gradually diffuses inward and downward from 10 s to 120 s. The temperature distribution on the side cross-section also intuitively confirms this upward-to-downward heating process, reflecting the collaborative process of heat transfer and material migration. It can also be seen that as heating time increases, the range of the high-temperature region continues to expand, and the temperature gradient gradually decreases. The cross-sectional results at different time points further reveal the spatial differences and evolution details of the internal temperature field, providing an intuitive basis for understanding water migration and redistribution in frozen soil under microwave action. The vertical temperature gradient is most pronounced in the early and middle heating stages, indicating that microwave energy decays rapidly with increasing penetration depth and leads to strong vertical non-uniformity of internal heat generation. With prolonged irradiation, the phase-change latent heat is gradually consumed, and thermal conduction between adjacent regions becomes more sufficient, which weakens local hot spots and promotes a more gradual temperature distribution inside the sample.

**Figure 12:**
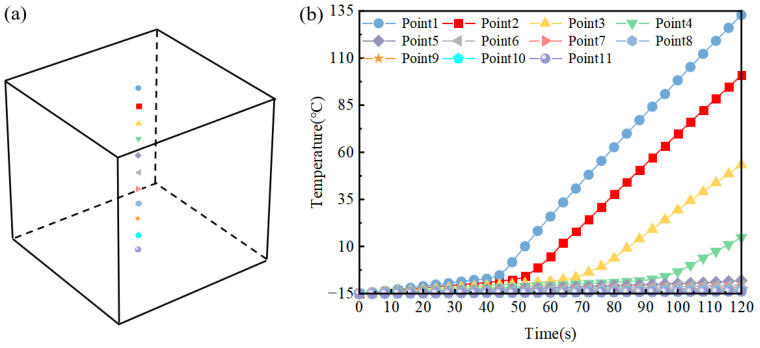
Variation in temperature inside frozen soil with time: (**a**) plan of observation points; (**b**) graph showing the variation in temperature at different observation points over time.

Figure 12a shows the plan view of the 11 observation points for the temporal variation in internal temperature in frozen soil. The 11 measurement points are in strict spatial correspondence with the 100 mm × 100 mm × 100 mm cubic frozen soil specimen. All measurement points are uniformly distributed along the central vertical axis of the specimen, with a vertical spacing of 10 mm between adjacent points, and are fixed at the geometric center of the horizontal cross-section of the specimen (with constant x and y coordinates of 50 mm). Among them, Point 1 precisely corresponds to the center of the specimen’s top irradiated surface directly facing the microwave waveguide port, and Point 11 corresponds to the center of the specimen’s bottom surface away from the microwave irradiation source, achieving full coverage of the entire vertical profile from the microwave-irradiated surface to the bottom of the specimen. This arrangement eliminates interference from the specimen’s boundary heat dissipation and electromagnetic field boundary reflection, enables accurate capture of the attenuation law of 2.45 GHz microwave energy with depth, and intuitively verifies the core characteristic of strong vertical non-uniformity in microwave thawing of frozen soil, namely, “preferential surface warming and rapid energy attenuation with depth”. Meanwhile, it provides boundary interference-free core measured data for the high-precision validation of the multi-physics coupling numerical model established in this paper and supports the core conclusions regarding the effective penetration depth of microwaves and frequency-band selection for engineering applications. (b) Temperature–time variation curves for corresponding measurement points: the horizontal axis represents the microwave irradiation duration ranging from 0 to 120 s, while the vertical axis denotes the thermodynamic temperature spanning from −15 °C to 135 °C. The curves distinctly reveal two core characteristics of microwave heating in frozen soil—Stagewise temperature rise: There is a pronounced phase in the heating process. During the initial 0–40 s, the temperatures at all measurement points remain stable within the −15 °C to −5 °C range, showing no significant increase. During this period, the input microwave energy primarily serves to overcome the latent heat associated with the ice-water phase transition, exhibiting a clear thermal startup lag effect. After 40 s, the surface measurement points first transition into a rapid heating stage. Strong vertical spatial non-uniformity: The temperature field exhibits a marked vertical gradient. Point 1, located at the sample surface, experiences the fastest heating rate, reaching approximately 135 °C at 120 s. As the depth of the measurement points increases, the heating rate sharply attenuates. At 120 s, Points 2, 3, and 4 register temperatures of roughly 97 °C, 50 °C, and 10 °C, respectively. In contrast, the deeper points (Points 5 through 11) consistently maintain the initial frozen temperatures ranging from −15 °C to 0 °C throughout the entire 120-s irradiation cycle, showing no significant temperature rise. This observation intuitively confirms the non-uniform heating characteristic of microwave-thawed frozen soil, where the surface preferentially absorbs energy, and the energy rapidly attenuates with increasing depth. This provides direct experimental support for understanding the temperature field evolution mechanisms in microwave thawing of frozen soil and for optimizing related process parameters.

### 5.3. Electromagnetic Field Distribution and Water Concentration Field Evolution

**Figure 13:**
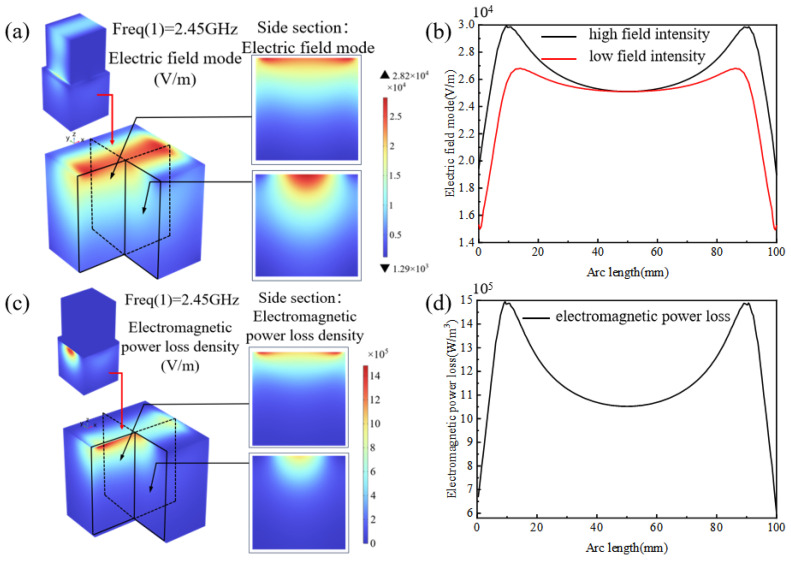
Electromagnetic-field characteristics in frozen soil: (**a**) electric-field intensity distribution; (**b**) electric-field intensity profile along the survey line; (**c**) electromagnetic power loss density distribution; (**d**) power loss density profile along the survey line.

Figure 13 illustrates the electromagnetic response characteristics within the frozen soil model. The propagation and distribution of electromagnetic waves inside the sample exhibit clear spatial non-uniformity: Along the measurement line, the electric field intensity reaches a peak at approximately 10 mm from the top surface, with a maximum value of 29.86 kV·m^−1^. The electromagnetic power loss density is primarily concentrated directly below the waveguide port and its adjacent region, being more pronounced at the relative position between the sample and the waveguide port, and gradually attenuating with distance from the active surface. Its peak also occurs at roughly 10 mm, with a maximum of 1.49 MW·m^−3^, aligning closely with the location of the electric field intensity peak, indicating a strong correspondence between energy deposition and field intensity distribution.

**Figure 14:**
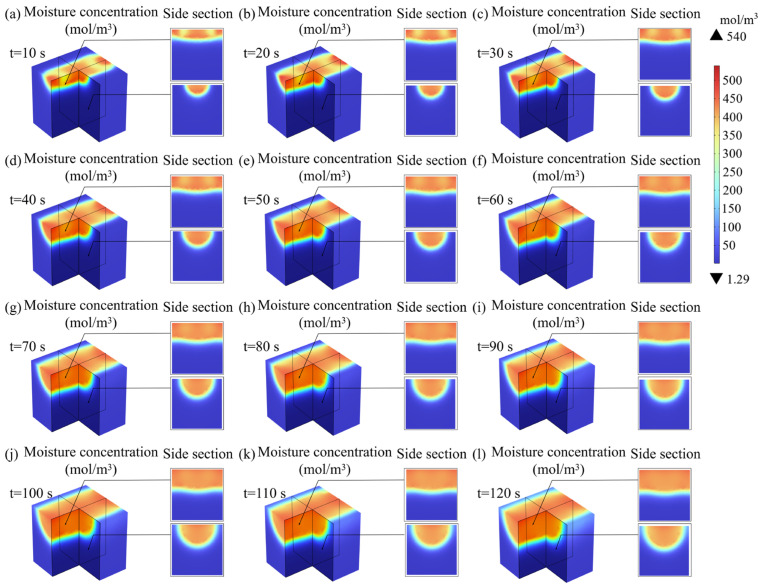
Distribution of water within permafrost over a period of 10 to 120 s (**a**) 10 s, (**b**) 20 s, (**c**) 30 s, (**d**) 40 s, (**e**) 50 s, (**f**) 60 s, (**g**) 70 s, (**h**) 80 s, (**i**) 90 s, (**j**) 100 s, (**k**) 110 s, (**l**) 120 s.

Figure 14 depicts the evolution of the water concentration field within frozen soil during microwave heating. Overall, regions of high-water concentration and temperature preferentially appear in the upper region of the cube and gradually diffuse inward and downward as heating progresses, reflecting the synergistic processes of heat transfer and material migration. Additionally, it can be observed that temperature rise accelerates the homogenization of the concentration field. Cross-sectional results under different initial conditions further reveal spatial differences and evolution details of internal concentration gradients, providing intuitive evidence for understanding water migration and redistribution in frozen soil under microwave influence.

## 6. Discussion

### 6.1. Mechanistic Interpretation of Core Experimental Findings

This study experimentally verifies the core hypothesis that gravimetric water content is the dominant factor controlling the dielectric response of frozen soil, and the frequency dependence of dielectric properties originates from the difference in polarization mechanisms of different phase components in frozen soil. Test results show that in the low-frequency band of 715–1150 MHz, the dielectric loss and water content sensitivity of frozen soil are significantly higher than those in the high-frequency band of 2250–2650 MHz. At 915 MHz, the real part of relative permittivity of frozen soil with 25% water content reaches 5.320, which is 1.88 times that of the sample with 15% water content, while this ratio is only 1.38 at 2450 MHz. Meanwhile, the microwave-induced temperature rise of frozen soil exhibits a robust three-stage characteristic, and the late-stage heating rate of the sample with low water content reaches 1.112 °C∙s^−1^, which is significantly higher than that of the high water content group.

The high dielectric response in the low-frequency band is dominated by the dipole orientation polarization of unfrozen water. The relaxation time of unfrozen water matches the alternating electric field period of the 915 MHz band, which maximizes the energy loss caused by the lag of dipole reorientation. In the 2450 MHz high-frequency band, the interfacial polarization of ice phase and soil skeleton and the lattice defect polarization become dominant, and the polarization contribution of unfrozen water is weakened, resulting in reduced sensitivity to water content. The physical essence of the three-stage temperature rise is the staged transformation of energy distribution: in the slow heating stage, microwave energy is mainly used for the initial temperature rise of unfrozen water and pre-melting of ice phase, in the isothermal platform stage near the freezing point, the input energy is completely consumed by the latent heat of ice-water phase change, with no sensible temperature rise, in the rapid heating stage, after the phase change is completed, all energy is used for the sensible temperature rise of liquid water and soil skeleton, and the sample with low water content has a higher heating rate due to its lower total heat capacity. In addition, the water vapor escape before the boiling point is driven by the pore water vapor pressure gradient generated by the temperature gradient. The measured pore water vapor pressure of 10–30 kPa in this study meets the critical migration pressure (≥10 kPa) for water vapor diffusion in frozen soil proposed by Zhang et al. [39], which explains the mechanism of water loss under non-boiling conditions. The higher the initial water content, the more uniform the pore water distribution, and the slower the accumulation of water vapor pressure, so the mass loss is negatively correlated with the initial water content.

This mechanistic interpretation directly responds to the core scientific question of this study by clarifying the dominant factors of dielectric response and the synergistic mechanism of electromagnetic–thermal–moisture coupling of frozen soil under microwave field and provides a theoretical basis for parameter optimization of microwave thawing.

### 6.2. Comparison with Existing Literature and Novelty of This Study

This study fills three key research gaps identified in the introduction, and the novelty is systematically verified through comparison with existing literature.

First, this study fills the gap of targeted experimental verification on the dielectric properties of frozen soil in 715–1150 MHz and 2250–2650 MHz dual bands, and clarifies the dominant polarization mechanism of different frequency bands. Bittelli et al. [6] only verified the feasibility of dielectric spectroscopy to estimate ice content in frozen soil, without distinguishing the difference of dominant mechanisms in high- and low-frequency bands. Jia et al. [15] only carried out microwave thawing experiments on frozen soil in a single 2.45 GHz band, without involving the comparison of dielectric properties in the 915 MHz industrial frequency band. Tarantino et al. [5] only focused on the correlation between dielectric constant and liquid water content, without quantifying the effect of frequency band on water content sensitivity. In this study, the dielectric parameters of dual bands were measured by coaxial probe method, and it was clarified that the low-frequency band is dominated by unfrozen water, and the high frequency band is dominated by ice phase and soil skeleton, and the water content sensitivity of frozen soil at 915 MHz is 1.36 times that at 2450 MHz. Most existing studies default 2.45 GHz as the industrial standard frequency band, ignoring the penetration advantage of 915 MHz in thick-layer frozen soil thawing. The dual-band experimental data in this study directly provide a quantitative basis for frequency-band selection in engineering: high water content frozen soil preferentially uses 915 MHz low-frequency microwave to match its deep penetration with high dielectric loss dominated by unfrozen water, low water content frozen soil uses 2450 MHz high-frequency microwave to improve heating efficiency through high power density. This conclusion cannot be provided by existing single-band studies, and improves the frequency dependence theory of frozen soil dielectric properties.

Second, this study clarifies the quantitative coupling relationship between the three-stage temperature rise of microwave thawing and moisture migration and mass loss, and improves the understanding of moisture migration mechanism during phase change in existing studies. Wei et al. [8] established a fully coupled electromagnetic–thermal–mass transfer model for microwave heating, but did not quantify the correlation between temperature rise stage and mass loss through experiments. Lindroth et al. [17] only focused on the engineering application effect of microwave thawing, without revealing the moisture migration mechanism during phase change. Zhang et al. [39] proposed the critical pressure of water vapor diffusion in frozen soil, but did not correlate it with the non-uniform temperature field of microwave heating. In this study, through 120 s continuous heating test, the heating rate, isothermal platform duration and mass loss of samples with different water contents were measured, the negative correlation between mass loss and initial water content was clarified, and the coupling driving mechanism of temperature gradient-vapor pressure gradient-moisture migration was verified. Most existing studies simplify microwave thawing as a pure heat conduction process, ignoring the reaction of moisture migration on temperature field distribution during phase change. This study confirms that even below the boiling point, water vapor migration driven by non-uniform temperature field will lead to mass loss, and the initial water content determines the degree of mass loss by regulating the accumulation rate of pore water vapor pressure. This mechanism explains the phenomenon of water content reduction at the edge of soil during microwave thawing in engineering, and provides a theoretical basis for water regulation during thawing.

Third, the three-dimensional electromagnetic–thermal–moisture migration coupling model established in this study uses the experimentally measured dielectric parameters as input, with a mean relative error (MRE) of only 4.72%, which is significantly better than existing empirical parameter models, and solves the problem that existing model parameters rely on empirical assumptions and have insufficient matching with experimental boundaries. Hansson et al. [29] established a hydro-thermal transport model for frozen soil, without considering the non-uniform distribution of electromagnetic field. The three-dimensional microwave heating model of Hong et al. [26] adopted empirical dielectric parameters, and the MRE with experimental results exceeded 10%. The coupled model of microwave heating of concrete by Wei et al. [8] did not optimize the parameters for the phase change characteristics of frozen soil. The model in this study adopts the measured dielectric parameters at 915 MHz and 2450 MHz, matches the boundary conditions of open microwave heating, and the MRE between the simulation results and the thermal imaging measured data is 4.72%, with a root mean square error (RMSE) of 3.85 °C, which meets the accuracy requirements of multi-physics coupling simulation in cold region geotechnical engineering (MRE < 5%). Existing microwave thawing models for frozen soil mostly use empirical dielectric parameters, ignoring the dynamic effects of water content and temperature on dielectric properties, resulting in large deviation between simulation results and experiments. The model in this study accurately reproduces the non-uniform distribution of electromagnetic field, the temperature field pattern of high temperature in the center, and the moisture migration law by introducing the measured dynamic dielectric parameters, and provides a high-fidelity numerical tool for engineering-scale microwave thawing simulation.

### 6.3. Geographical Applicability and Generalization of Findings

The core conclusions of this study have cross-geographical universality, and the application boundary is clearly defined, which can be adapted to frozen soil types in different cold regions through parameter correction.

The test soil sample in this study is taken from seasonally frozen silty clay in Hegang, Heilongjiang, with a plastic limit of 18.4% and a liquid limit of 30.6%, which is highly consistent with the physical indexes of silty clay and silt in the seasonal frozen soil region of Northeast China, the permafrost region of the Qinghai–Tibet Plateau, and the seasonal frozen soil region of the Loess Plateau in Northwest China. The core conclusions of this study: water content is the dominant factor of dielectric response, the frequency dependence of dielectric properties, and the three-stage characteristics of microwave heating, are all determined by the dielectric properties and polarization mechanism of the ice-water-soil skeleton three-phase in frozen soil, and are not directly related to the geographical origin of the soil.

For frozen soil in different geographical regions, the universality of the conclusions of this study is divided into the following three levels: (1) core mechanism level—the dominant factors of dielectric response and multi-field coupling mechanism are completely universal, applicable to all types of frozen soil in cold regions around the world. (2) Quantitative parameter level—the dielectric parameters, heating rate and thawing duration provided in this study are applicable to frozen soil with similar physical properties to Hegang silty clay. For high-salinity frozen soil, organic frozen soil and sandy gravel frozen soil, the dielectric parameters should be measured by the test method in this study to correct the model input. (3) Engineering application level—the frequency-band selection principle, hot spot suppression measures and thawing duration design method proposed in this study are applicable to all frozen-soil thawing projects in cold regions, and only need to adjust the process parameters according to the soil type.

## 7. Conclusions

This study systematically investigates the dielectric properties and electromagnetic–thermal–moisture coupling response of frozen soil under microwave irradiation via laboratory tests and numerical simulation. The core objective conclusions are as follows:

1. The dielectric response of frozen soil is dominated by gravimetric water content and exhibits significant frequency dependence. The real part of relative permittivity, dielectric loss factor, and loss tangent of frozen soil increase with rising water content. The low-frequency band (715–1115 MHz) is dominated by unfrozen water, with significantly higher dielectric loss and water content sensitivity than the high-frequency band (2250–2650 MHz), which is governed by ice phase and soil matrix.
2. The temperature rise process of frozen soil under microwave irradiation presents a consistent three-stage characteristic: slow temperature rise, isothermal plateau near the freezing point, and rapid temperature rise. Specimens with lower initial water content have higher temperature rise efficiency in the late heating stage, while specimens with higher initial water content achieve greater total temperature rise over the full irradiation period.
3. Moisture mass loss of frozen soil during microwave thawing is negatively correlated with initial gravimetric water content. Moisture migration and vapor exfiltration occur before the specimen reaches the atmospheric boiling point, which is co-driven by temperature gradient and vapor pressure gradient within the frozen soil pores.
4. The spatial non-uniformity of the microwave electromagnetic field leads to a distinct temperature field pattern with high temperature at the specimen center and low temperature at the edges, with a maximum center-to-edge temperature difference of 45–60 °C.

## Figures and Tables

**Table 1:** The dielectric properties of the material at 915 MHz and 2450 MHz.

Frequency	Material Under Test	Complex Relative Permittivity	Loss Tangent (tan δ)
915 MHz	soil	3.896 + 0.730 j	0.187
	ice	3.187 + 0.427 j	0.115
	water	80.451 + 3.978 j	0.049
	S15 frozen soil	2.835 + 0.431 j	0.152
	S20 frozen soil	3.662 + 0.696 j	0.184
	S25 frozen soil	5.320 + 0.712 j	0.191
2450 MHz	soil	3.310 + 0.542 j	0.164
	ice	1.852 + 0.222 j	0.120
	water	79.106 + 10.544 j	0.133
	S15 frozen soil	2.422 + 0.328 j	0.133
	S20 frozen soil	2.500 + 0.477 j	0.156
	S25 frozen soil	3.346 + 0.617 j	0.184

Table Note: j is the imaginary unit widely used in RF and microwave engineering with j^2^ = −1.

**Table 2:** Simulation parameter table.

Parameter Type	Value/Expression	Source
microwave frequency	2.45 GHz	Common industrial microwave frequency
microwave power	1.5 kW	Matching the set power in the microwave heating experiment
permeability	1.3 × 10^−10^ $m^{2}$	Wang, Q. L., Chen, L., Ming, F. [38]
specific heat capacity of water	4182 $J\cdot {\left(kg\cdot K\right)}^{-1}$	Wang, J., Deng, J., Zheng, J., Wang, T., Yu, Y. [30]
thermal conductivity of ice	2.14 $W\cdot {\left(m\cdot K\right)}^{-1}$	Wang, J., Deng, J., Zheng, J., Wang, T., Yu, Y. [30]
specific heat capacity of ice	2060 $J\cdot {\left(kg\cdot K\right)}^{-1}$	Wang, J., Deng, J., Zheng, J., Wang, T., Yu, Y. [30]
latent heat of ice-water phase change	334 $kJ\cdot kg^{-1}$	Wang, J., Deng, J., Zheng, J., Wang, T., Yu, Y. [30]
dynamic viscosity of water	1.793 × 10^−3^ Pa⋅s	Liu, X. Q., Zhang, J. G., Fan, L. P. [36]
density of water	1000 $kg\cdot m^{-3}$	Standard density of water at normal temperature and pressure
density of ice	920 $kg\cdot m^{-3}$	Standard physical density parameter of ice

**Table 3:** Data of typical measuring points.

Time	Category	Center	Top Left	Top Right	Bottom Left	Bottom Right
30 s	Experiment	42.3	18.6	17.9	18.2	17.5
	Simulation	38.7	16.2	15.8	16	15.3
60 s	Experiment	98.5	45.2	44.3	44.8	43.9
	Simulation	92.1	41.6	40.9	41.3	40.5
90 s	Experiment	143.2	80.6	79.7	80.1	79.3
	Simulation	136.7	76.2	75.4	75.8	75
120 s	Experiment	151.2	87	86.1	86.5	85.7
	Simulation	145.3	82.9	82.1	82.5	81.7

## Data Availability

The data presented in this study are available upon reasonable request from the corresponding author.
